# Principal component analysis of the relationship between pelvic inclination and lumbar lordosis

**DOI:** 10.1186/s13013-019-0175-5

**Published:** 2019-02-13

**Authors:** Geoff Dakin, Raymond J. Turner, Stephana J. Cherak

**Affiliations:** 1 Dakin Rehab Inc., #603-550 11 Ave SW, Calgary, Alberta T2R 1M7 Canada; 2 Department of Biological Sciences, University of Calgary, Calgary, Alberta T2N 1N4 Canada; 3 Alberta Children’s Hospital Research Institute for Child and Maternal Health, Heritage Medical Research Building, 3330 Hospital Drive NW, Calgary, Alberta T2N 4N1 Canada

**Keywords:** Pelvic inclination, Pelvic tilt, Pelvis, Lumbar lordosis, Spine, Surface topography

## Abstract

**Background:**

The purpose of this study was to describe the relationship between pelvic inclination (PI) and lumbar lordosis (LL). Pelvic inclination and pelvic tilt are two different names for the same metric. The geometrical parameters of the spine and pelvis were measured using surface topography scanning, and the data was explored for any physical relationships using principal component analysis.

Once widely assumed to be a direct correlation, research in the 1980s first cast doubt upon the PI to LL relationship. And yet, other studies have suggested a relationship does exist. Decades later, the rehabilitation professionals often still rely on this supposed correlation when making decisions about rehabilitation treatment interventions. This theoretical relationship requires further clarification, which is explored herein.

**Methods:**

Surface topography imaging is a technology that has proven to be a radiation-free way to produce accurate, reliable skeletal alignment measures. Patient data from one physical rehabilitation clinic was collected at the time of initial assessment. Patients presented with a wide range of musculoskeletal complaints. Surface topography scans were performed on 107 patients at the commencement and completion of their therapy. Principal component analysis was performed on the collected data to determine how these spine and pelvic alignment parameters changed between the two points in time and what trends and/or relationships exist between the parameters. Our analysis evaluated eight spinal and pelvic measurements as input and focused on LL and PI as the two principal components at time points of beginning and completion of treatment.

**Results:**

Pelvic inclination and lumbar lordosis changed during treatment but were not correlated.

**Conclusion:**

Our data demonstrates that pelvic inclination and lumbar lordosis do not have a predictable relationship as previously assumed.

## Highlights

- Empirical evidence, such as the use of the pelvic tilt exercise, appears to support a relationship between PI and LL.
- Research data exists that supports a relationship between PI and LL while other data does not.
- We quantitatively analyzed the correlation PI and LL.
- We found no correlation between PI and LL.
- Further studies (using a larger cohort size?) are encouraged to refine our understanding of this relationship. What conditions have been present in the studies that have suggested a relationship exists?

## Introduction

Healthcare providers and patients alike remain frustrated about the causes of postural imbalance, and effective long-term treatment is rendered to recurring estimations. However, strong correlations between certain pelvic and spinal positional parameters for a specific patient in an upright posture have been described [6, 8]. Unfortunately, there is still a paucity of information on how common spinal and pelvic parameters correlate and shift through the effect of treatment, yielding little information as to the best-practice method of malalignment treatment [5].

The current gold standard for diagnosis and subsequent surveillance remains a standing full-column radiographs of the spine; however, and especially so in young patients, repeated exposure to ionizing radiation causes a significant increase in the risk of malignancies later in life [1]. Surface topography (ST) has been developed as an alternative to plain radiographs and has been determined as a suitable replacement [2, 4]. This technology has proven to provide a safe, accurate, and affordable alternative to traditional X-ray assessment.

Here, we mined a data set from ST analysis of patients who presented with a wide range of musculoskeletal complaints and postural imbalance. Patients were assessed before and after a course of treatment consisting of soft tissue manipulation and corrective exercise. Data mining performed independently of the clinical practice and changing protocols has great potential for exploring the hidden patterns in medical data collections and finds its application in almost all areas of biomedicine [2]. These patterns can then be utilized for clinical diagnosis and better understanding of disease and dysfunction.

Principal component analysis (PCA) is commonly used to transform complex and multivariate feature space to a smaller and more meaningful representation to observe any correlations. Our study evaluated various pelvis and spine alignment parameters and exploited PCA transformation to visualize variations and correlations among spinal and pelvic features. It is important to note that we were not attempting to assess the efficacy of the therapeutic intervention but only the amount of alignment change in the spine and pelvis, then determine what relationships might exist between those alignment parameters. The primary aim of this work was to investigate the relationship between pelvic inclination and lumbar lordosis. A more thorough understanding of the correlations between skeletal alignment parameters should enable clinicians to better understand and treat biomechanical dysfunction in the body.

## Materials and methods

### Surface topography measurements

Data from one physical rehabilitation clinic was utilized. Patients were referred to the clinic with various musculoskeletal issues arising from malalignment problems with the pelvis and spine. Surface topography measurements of patients were obtained using surface topography scanning (DIERS Formetric, Diers Medical Systems, Chicago, IL). Two ST scans were obtained for each patient: one at the commencement and one at the completion of a 12-week therapy treatment protocol involving individually prescribed manual therapy and corrective exercise. Data from 107 patients was utilized that had completed the treatment with scans prior and post-treatment. No patient identifier information was collected. The data analysis was performed post-patient treatment, and therefore information from this study was not used to alter patient treatment. The treatment in these cases involved manual manipulation of soft tissues and a personally prescribed daily corrective exercise routine designed to progressively move the patient away from malalignment.

To obtain the scan, the patient stood in an upright position at a distance of 2 m in front of the ST scanner. The scanner projects white light raster lines onto the back of the standing patient, taking two pictures per second over a 6-s period. Each digital image is then obtained and assessed by computational algorithms, and a total of 12 images are evaluated and averaged by the instrument’s software. A three-dimensional (3D) image of the spine is created from ST results.

**Fig. 1:**
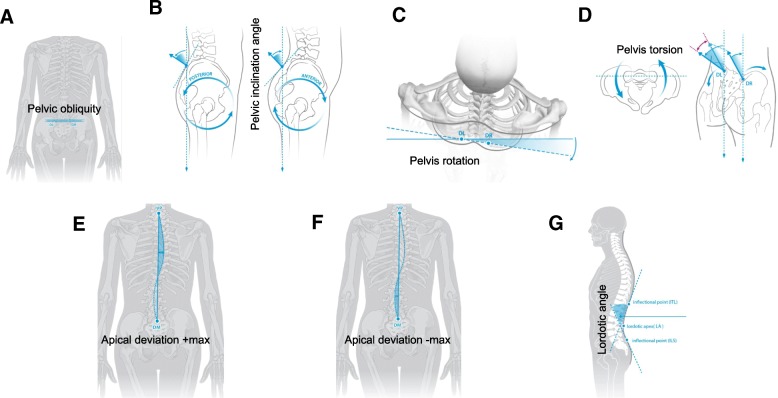
Schematic diagram of spinal and pelvis measurements. **a** Pelvic obliquity, PO; **b** pelvic inclination angle, PIA; **c** pelvis rotation, PR; **d** pelvis torsion, PT; **e** apical deviation +max, AD+; **f** apical deviation –max, AD−; **g** lordotic angle, LA

It is important to note that ST measures kyphosis and lordosis using the surface tangents of the inflection points of the convex and concave curves, and pelvic obliquity (PO) is measured using topographical landmarks such as the vertebral prominence of C7 and the dimples created by the posterior superior iliac spines of the ilia. This analysis provides the following skeletal measurements: pelvic obliquity (PO), pelvic inclination angle DL (PIA DL), pelvic inclination angle DR (PIA DR), pelvis rotation (PR), pelvic torsion (PT), apical deviation +max (AD+) [deviation to the right], apical deviation –max (AD−) [deviation to the left], and lordotic angle (LA). A schematic diagram depicting rough skeletal measurements is provided for in Fig. 1. The descriptive parameters were then calculated with mean and standard deviation for continuous variables and are provided for in Table 1.

**Table 1:** The reference basis for the positional and morphological parameters in 107 normal subjects at the commencement and completion of treatment

Input values		Mean			SD		
Name	Abbrv.	Total	Start	Final	Total	Start	Final
Pelvic obliquity	PO	2.50	2.29	2.70	1.86	1.76	1.97
Pelvic inclination angle DL	PIA DL	20.60	20.51	20.70	6.81	7.07	6.56
Pelvic inclination angle DR	PIA DR	20.64	20.41	20.87	6.96	6.80	7.12
Pelvis rotation	PR	2.36	2.36	2.36	1.68	1.75	1.60
Pelvis torsion	PT	2.26	2.31	2.20	2.26	2.51	1.99
Apical deviation +max [right]	AD+	7.37	7.59	7.15	5.14	4.90	5.32
Apical deviation –max [left]	AD−	5.31	5.00	5.64	4.38	2.83	3.85
Lordotic angle	LA	40.46	42.17	43.29	9.92	37.73	8.32

### Statistical analysis

All data processing was performed by an individual blinded of patient symptom display and result expectation. The principal component analysis was conducted using the RStudio package (RStudio, Inc., Boston, MA). Statistical calculations were performed using Prism 7.0 (GraphPad Software, Inc.). Grubb’s test was conducted on all data to determine statistical outliers (*P* < 0.05).

## Results

### Population

Data from 107 (*n* = 107) patients who had visited the clinic and received treatment between December 2016 and March 2017. Patients initially presented with a wide variety of musculoskeletal complaints, with 100% exhibiting postural imbalance. Patients were scanned prior to treatment and again on subsequent visits to provide post-treatment data.

### Distribution of the data

**Fig. 2:**
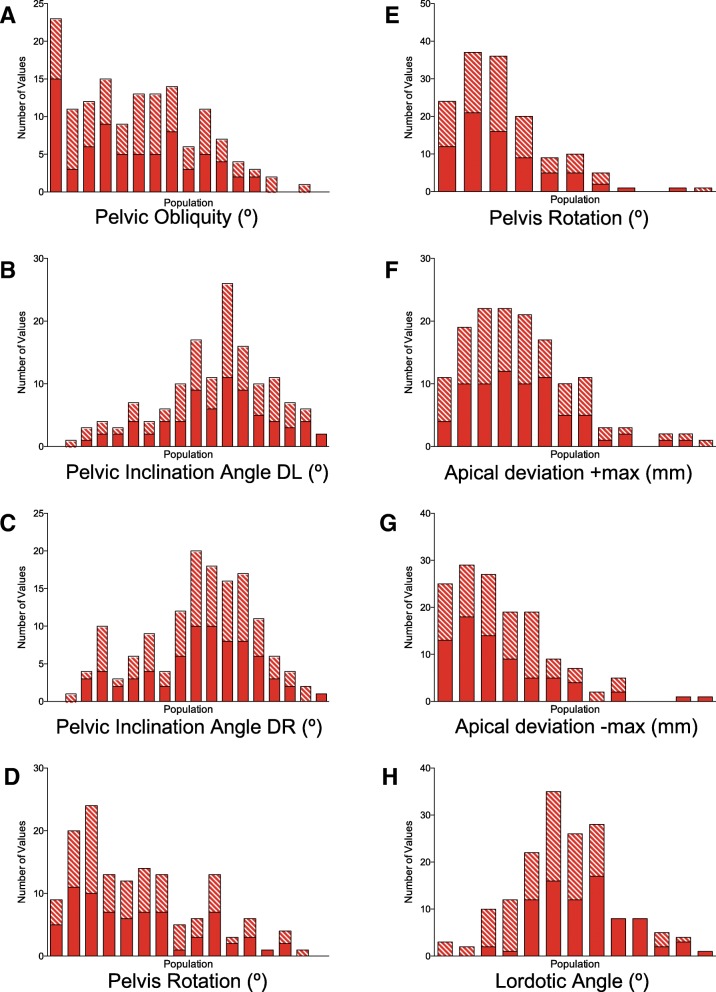
Distribution plots of the eight input variables at the commencement (solid) and completion (hashed) of malalignment treatment. Populations along the abscissa were binned according to crude input values. **a** Pelvic obliquity, PO; **b** pelvic inclination angle DL, PIA DL; **c** pelvic inclination angle DR, PIA DR; **d** pelvis rotation, PR; **e** pelvis torsion, PT; **f** apical deviation +max, AD+; **g** apical deviation –max, AD−; **h** lordotic angle, LA. The average result of eight input variables are provided for in Table 1

Spine and pelvis positional and morphological parameters are recorded in Table 1 for the patient cohort. Patient datasets were subsequently binned according to the value of the measurement; binning a data set is a process of grouping measure data into data classes of similar value ranges to be used in various analyses. With the data now binned, the data can be evaluated for statistical profiling of the data. Shown in Fig. 2 is the plotted binned data subjected to the D’Agostino-Pearson normality test, for which normality was satisfied for all the pelvic and spinal parameters (Fig. 2) (i.e., no statistically significant outliers). The distribution of values at the commencement and completion of treatment, in addition to the degree of change as an effect of treatment duration, are observed in Fig. 2. Neither the distribution nor the precision of the count data changed from commencement to completion for any parameter.

### Principal component analysis

In datasets containing many input variables such as this, one of the inherent difficulties is visualizing the data to find trends. This is because groups of variables often move together and may measure the same driving principal governing the behavior of the system. The redundancy of this information can be simplified by replacing a group of variables with a single new variable. The myriad of variables are reduced to a few, interpretable linear combinations, where each linear combination will correspond to a particular principal component. A line segment plot showing the fraction of total variance in the data is subsequently produced; a scree plot. Each principal component (PCn) represents the underlying variables where there is most variance in the data, which are across the abscissa axis. Now, with the data grouped into components, examining plots of these few new variables can develop a deeper understanding of the driving forces that generated the original data.

Here, as the null hypothesis, the eight input variables of PO, PIA DL, PIA DR, PR, PT, AD+, AD−, and LA were hypothesized to be interconnected and affect each other. The principal components were computed using the variance technique to produce a scree plot. Before the commencement of treatment, a large portion of the variance (43.5%) in the data is shown by the first two components; the first component accounts for 24.7%, and the second component accounts for 18.8% in whole data. At the completion of treatment, two factors were as well extracted from the scree plot (Fig. 2b) with a cumulative variance of 47.2%; the first component accounts for 26.8%, and the second component accounts for 20.4% in the whole data. For simplicity of analyses herein, PC1 and PC2 were found to be most dominant for measurement parameter differences at both time periods; PC3 through PC8 were found not to add any further interpretation to the hypothesis and were thus not used. Thus, with the PCA, eight input features were now replaced by two principal components at both time points (i.e., a large set of variables was reduced to a small set of variables that still contained most of the information in the large set).

In PCA, the original data is transformed to a new coordinate system such that the greatest variance by any projection of the data comes to lie on the first coordinate (PC1) and the second greatest variance on the second coordinate (PC2). Conclusions about the subsequent correlations can be drawn. The variables located in the same quadrant are said to be correlated and move together. The variables that are located in adjacent quadrants have no effect on each other, and the variables in the opposite quadrant are negatively correlated to each other; if one increases the other decreases. Here, PCA was utilized to provide a portal to understand the complex relationships toward a visual representation of pelvic and spinal parameter variations.

**Fig. 3:**
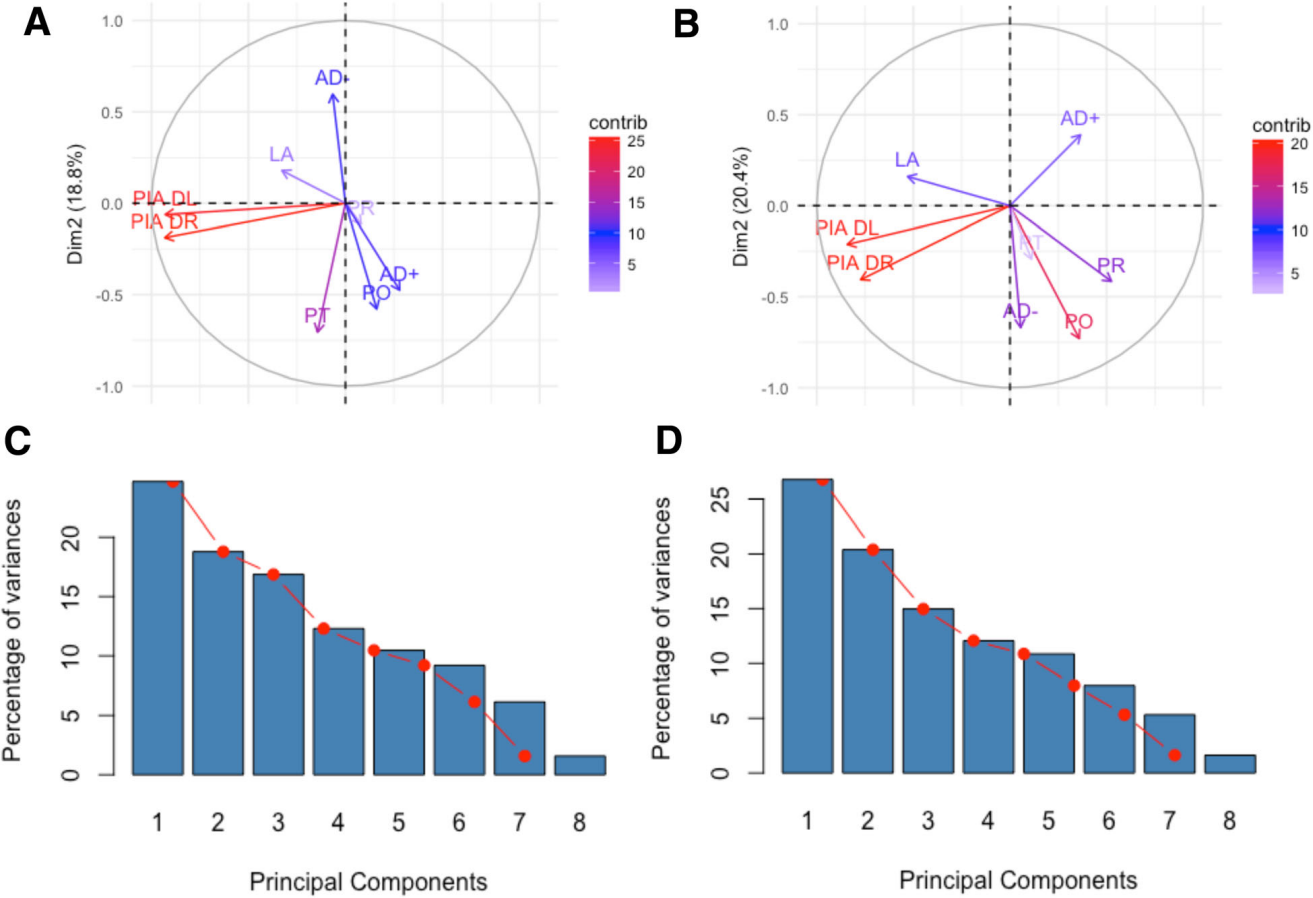
Plot of correlated variables at the commencement (**a**) and completion (**b**) of treatment by the PC1 and PC2. Pelvic obliquity, PO; pelvic inclination angle DL, PIA DL; pelvic inclination angle DR, PIA DR; pelvis rotation, PR; pelvis torsion, PT; apical deviation +max, AD+; apical deviation –max, AD−; lordotic angle, LA. Plots illustrate the magnitude of input value correlation respective to the PC1 and PC2 obtained at the commencement and completion of malalignment treatment; the contribution of PC1 and PC2 to total variance is illustrated in the scree plots

Prior to the commencement of treatment, PIA DL, PIA DR, and PT lie in the same quadrant and are therefore correlated as expected (Fig. 3a). PO, PR, and AD+ were also found to be correlated; however, these did not correlate with any other spinal parameters. Moreover, the magnitude of the lines in Fig. 3—schematically shown by color—gives the significance of the respective variables. It can be seen from Fig. 3a that PR is the least significant variable. It was unexpected that prior to treatment, the variables of PIA DL, PIA DR, and PT did not display a negative correlation with any of the other parameters. However, AD+ has a negative correlation with AD− as was hypothesized. Most interestingly, PIA DL, PIA DR, and PT were not correlated to LA; the most surprising was a lack of relationship between PIA DL/DR and LA as is currently widely assumed. On the other hand, AD− indeed showed a correlation with LA.

In Fig. 3b, we see that PIA DL and PIA DR continued to show the strongest correlation and contribution at the completion of treatment; PT showed a markedly reduced contribution and became correlated to AD−, PO, and PR. Interestingly, the contribution of PR was only significant at the completion of treatment and did not show significance at the commencement, but the positioning of the vector remains the same. Moreover, the movement of AD−, AD+, PT, and PR resulted in a correlation between all of the aforementioned variables. Whereas AD+ and AD− were negatively correlated prior to treatment, at the completion, this relationship was lost. Furthermore, while a similar contribution was shown between AD+ and AD− prior to treatment, at the completion the contribution of AD− was increased relative to AD+. The LA was not correlated to any other input variable at the completion of treatment and was negatively correlated to AD−, PT, PO, and PR.

## Discussion

The human spine is a multifunctional structure of the human body comprised of the bones, joints, ligaments, and muscles. It is widely believed that poor alignment of a person’s bones can interrupt healthy function of joints and can cause neuromuscular compensations elsewhere in the body. Joint centration is a term that implies proper joint alignment; malalignment implies the opposite. Treatment of postural imbalance issues of the spine and/or pelvis is a complex process without a “best practice” firmly established. According to the United States Department of Labor Statistics, there are 210,900 physical therapists, 45,200 chiropractors and 168,800 massage therapists (as of 2014) in the USA alone. Within this community of practitioners and beyond (i.e., personal trainers, athletic trainers, interested members of the general public), it is widely believed that there is a direct correlation between PI (i.e., pelvic inclination/tilt) and LL (i.e., lumbar lordosis/lordotic angle). This belief is so widely assumed that it is factored into the treatment and corrective exercise decisions of hundreds of thousands of practitioners and therefore influences the health and wellbeing of tens of millions of people in the USA alone. Is this biomechanical relationship real or imagined? Surprisingly this question has received little attention from the research community over the years. Moreover, the research that has cast doubt upon this assumed biomechanical relationship [10] has not seemed to change common clinical practices. If this assumed biomechanical relationship is a mirage, surely a scientific explanation would be invaluable. This situation as it currently exists does a disservice to the healthcare community and to the public as well. Our study utilized surface topography scanning and principal component analysis to examine the relationships between eight specific spine and pelvic alignment parameters, including (and for our purposes, most importantly) PI and LL.

### Effect of treatment on spine and pelvis input variables

In this study, standard references of pelvic and spinal parameters were assessed whose anthropometrical parameters were found to be in a normal distribution. The normality distribution was as well accepted for all pelvic and spinal parameters. Our mean standard deviation of spinal and pelvic parameters is comparable to the background data in an adult control population [3, 7–9].

### Relationship between spine and pelvis input variables

To deal with the complex data set, PCA was successfully applied for the visual analysis of variations in pelvic and spinal parameters. This 2D representation of multivariate data proved easy to understand and provided meaningful information. The correlations between PIA DL, PIA DR, AD+, AD−, PO, PR, PT, and LA were explored at the commencement and completion of postural imbalance treatment. Some interesting patterns were observed from multivariate analysis. At both the commencement and completion of treatment, PIA DL was found to have a very strong correlation with PIA DR and there was no correlation of either variable with the LA. At the commencement, PO and AD+ also showed a strong correlation with AD− being negatively correlated. Conversely, at the completion of treatment, PO was correlated with AD−, while AD+ was not with either. PT and PR exhibited the largest change in contribution between the start and end of treatment as illustrated by their vector magnitudes and their contribution (Fig. 3).

The ways of adaptation between the spine and pelvis are unbalanced and can improve pathological patterns on a long- or short-term basis. If, for example, the measured lordosis is out of the confidence limits of the predicted lordosis range, the standing posture is no longer in the conditions of the value of biochemical economy: the adaptation potential of the spine and pelvis is exceeded. A future randomized, controlled study should investigate whether our finding that PIA and the LA do not correlate, and especially so with the duration of treatment, accurately reflects what will happen in a greater population. Conceivably, evaluating patients through longitudinal assessments over time could yield valuable clinical information.

## Conclusions

We are not the first to present data that disputes that PI and LL are correlated. However, here we come to that conclusion utilizing surface topography technology. Furthermore, given that earlier research revealed these findings and the physical rehabilitation community has not widely responded with clinical practices reflecting this “new” information suggests that this topic demands more discussion and investigation.

## Abbreviations

3D: Three dimensional
AD−: Apical deviation −max
AD+: Apical deviation +max
LA: Lordotic angle
LL: Lumbar lordosis
PCA: Principal component analysis
PCn: Principal component
PI: Pelvic inclination
PIA DL: Pelvic inclination angle DL
PIA DR: Pelvic inclination angle DR
PO: Pelvic obliquity
PR: Pelvis rotation
PT: Pelvis torsion
ST: Surface topography
